# Role of Resveratrol on Indoxyl Sulfate-Induced Endothelial Hyperpermeability via Aryl Hydrocarbon Receptor (AHR)/Src-Dependent Pathway

**DOI:** 10.1155/2019/5847040

**Published:** 2019-11-27

**Authors:** Eskedar Getachew Assefa, Qiaoqiao Yan, Siameregn Berhe Gezahegn, Maibouge Tanko Mahamane Salissou, Shuiqing He, Nannan Wu, Xuezhi Zuo, Chenjiang Ying

**Affiliations:** ^1^Department of Nutrition and Food Hygiene, Hubei Key Laboratory of Food Nutrition and Safety, School of Public Health, Tongji Medical College, Huazhong University of Science and Technology, Wuhan 430030, China; ^2^Department of Food Science and Applied Nutrition, Addis Ababa Science and Technology University, P.O. Box 16417, Ethiopia; ^3^Department of Clinical Nutrition, Tongji Hospital, Huazhong University of Science and Technology, Wuhan, 430030 Hubei, China; ^4^Sport Science Academy, Kotebe Metropolitan University, Addis Ababa, Ethiopia; ^5^Department of Pathology Pathophysiology, School of Basic Medicine, Key Laboratory of Education Ministry of China for Neurological Disorders, Tongji Medical College, Huazhong University of Science and Technology, Wuhan 430030, China; ^6^Ministry of Education Key Lab of Environment and Health, School of Public Health, Tongji Medical College, Huazhong University of Science and Technology, Wuhan 430030, China

## Abstract

Resveratrol (RES), a dietary polyphenol compound, has been shown to possess health benefits due to its anti-inflammatory, antioxidative, and antiatherosclerosis properties. Tryptophan metabolite-derived indoxyl sulfate (IS) is identified as one of the uremic toxins and physiological endogenous ligand/activator of aryl hydrocarbon receptor (AHR), associated with atherosclerosis in chronic kidney disease (CKD) patients. Studies have shown that a high serum level of IS causes deleterious effects on health primarily by inducing oxidative stress and endothelial dysfunction. However, the precise mechanisms are still unclear. Here, we investigated the underlying mechanism of IS effect on endothelial permeability and the role of RES on IS-induced endothelial hyperpermeability via the AHR/Src-dependent pathway. Bovine aorta endothelial cells (BAECs) were cultured and incubated with IS in the presence or absence of RES, and transendothelial electrical resistance (TEER) and permeability of cells were measured. Alongside, AHR, Src kinase, and Vascular Endothelial Cadherin (VE-Cadherin) activation were examined. Our data showed that IS reduced TEER of cells resulting in increased permeability. VE-Cadherin, a vital regulator of endothelial permeability, was also significantly activated in response to IS, which appeared to be associated with changes of endothelial permeability and AHR/Src kinase. Interestingly, in this setting, RES reversed the effect of IS and inhibited the increased activation of Src induced by IS-activated AHR and modulated VE-Cadherin and permeability. CH223191, an inhibitor of AHR, significantly inhibits IS-induced endothelial hyperpermeability. Further analysis with treatment of PP2, an inhibitor of Src abolishing Src activation, suggests downstream factors. All our data indicated that IS upregulated the AHR/Src kinase pathway, and increased endothelial permeability and phosphorylation of VE-Cadherin may be represented and provide new strategies for addressing protective properties of RES against Src kinase involved in AHR-mediated endothelial hyperpermeability. The findings may be crucial for managing diseases in which endothelial permeability is compromised, and the dietary polyphenols are involved.

## 1. Introduction

The endothelium has exponentially been identified as a semipermeable barrier and an essential regulator of blood flow in both micro- and macrovascular beds. The endothelium lining the intima of the blood vessels plays crucial roles in the homeostasis of a variety of functions such as vascular smooth muscle tone, angiogenesis, host-defense reactions, and tissue fluid hemostasis [1]. The maintenance of a semipermeable barrier by the endothelium is remarkably essential in controlling the passage of fluid along with macromolecules between interstitial space and blood. It has been documented that loss of this function results in increased permeability [2]. Endothelial hyperpermeability shared characteristics of many diseases, including atherosclerosis trauma, sepsis, diabetes mellitus, uremic syndrome, and tissue inflammation, and hallmark of inflammatory diseases such as the acute respiratory distress syndrome [3–6]. The permeability characteristics of transported macromolecules are associated with their molecular radii together with the barrier properties of the specific endothelium [7].

Due to lack of renal clearance in CKD patients, retention/accumulation of uremic solutes is increased in proximal tubular cells and causes most of the renal injury and also contributes to elevated serum levels of uremic toxins [8]. Uremic toxin accumulation in serum under pathological condition is a primary event during the formation of atherosclerosis [9]. IS is a uremic toxin derived from a metabolite of dietary protein (tryptophan) and a risk factor for aggravating cardiovascular disease (CVD) in CKD patients mainly due to inducing oxidative activity and endothelial dysfunction [10, 11]. Intestinal bacteria form the precursor of IS via tryptophan degradation before absorption [12, 13]. Following ingestion of dietary protein, part of protein-derived tryptophan is cleaved into indole by the action of tryptophanase colonic microbes such as Escherichia coli and the indole is absorbed from the intestine into the blood and oxidized to indoxyl in the liver by the action of cytochrome p450 enzyme, and the indoxyl is further sulfonated in the liver and forms IS [14]. Recent clinical studies revealed that IS, a protein-bound indole derivatives uremic toxin, is one of risk factor for cardiovascular death in CKD patients (stage 2 to hemodialysis stage 5) [15] and considered major causes with an estimated 20-fold in cardiovascular mortality [16]. High serum concentration of IS is increased in CKD patients which primarily affect renal function and contributes to CKD progression which may also be recognized as the major cause of impairment of endothelial function, an initial step for atherosclerosis that accelerates CVD progression in CKD patients [11, 15]. The increased concentration of IS may result in an upsurge in the generation of free radicals and activate proinflammatory factors, leading to the progression of CVD. Studies revealed that IS-enhanced vascular injury exhibits inflammatory effects and impaired endothelial healing ability [17, 18]. In addition, IS is one of endogenous physiological ligands of AHR among indole derivatives such as tryptophan, indole, indoxyl-3-sulfate (I3S), indoxyl-3-acetate (I3A), and indole-3-methanol and a photoproduct of tryptophan through structural and chromatographic studies and characterized as potent agonists and activator of AHR [19, 20], and studies suggest that ligand-activated AHR induces a response in cardiovascular cells, although there are other potential agonists that directly stimulate AHR [21].

Ligand-AHR activation has been involved in the development of impaired endothelial function leading to endothelial cell dysfunction and injury, followed by the development of CVD [22]. AHR belonging to the basic helix-loop-helix/Per-ARNT-Sim (bHLH-PAS) superfamily is activated by numerous endogenous and exogenous ligands and characterized as a ligand-dependent transcription factor [23]. Prior to ligand binding, AHR is located in the cytoplasm, a complex with different proteins such as heat-shock protein 90 (Hsp90), Hsp90-interacting protein P23, immunophilin-like protein XAP2 (AIP or ARA9), and Src kinase, and could control cellular processes along with other transcriptional factors in the cytoplasm [24–26]. Previous studies revealed that AHR function is mediated by different signaling pathways such as genomic and nongenomic pathways. The AHR genomic pathway is also called AHR canonical pathway. In AHR genomic pathway, the ligand enters the plasma membrane via passive diffusion and binds/activates AHR in the cytoplasm. Upon binding, ligand-AHR complex is exposed to nuclear translocation and forms a heterodimeric complex with its partner AHR nuclear translocator (ARNT also called HIF-1b) [27]. The heterodimer (AHR-ARNT) binds to DNA specific sequence named xenobiotic response elements (XREs) or dioxin response elements (DRE) located in the promoter region of target genes and induces the transcription of target genes (CYP1A1) and inflammatory response [23, 28, 29]. In classical AHR genomic pathway, the AHR activation is a ligand-dependent transcription factor. However, AHR also regulates gene expression through nongenomic, ARNT-independent genomic, as well as nonclassical, signaling pathway [26, 30]. Recently, it was reported that AHR regulated the number of genes without direct binding of the AHR to DRE consensus sequence [31].

In AHR nongenomic pathways, ligand-activated AHR leads to Src kinase activation and allows the dissociation from the AHR receptor complex in the cytoplasm and translocates to the membrane [32]. In the normal and pathological processes, Src kinase is known to regulate intracellular signal transduction in vascular biology, including endothelial cell permeability [33]. The integrity of cell to cell contact barrier function is disturbed by the activation of the Src kinase induced following the exposure to ligands [22]. Src kinase plays a role in regulating cadherin-dependent cell-cell contact accomplished by VE-Cadherin endothelial permeability [34]. In endothelial cells, VE-Cadherin is the most crucial cell-specific member of the cadherin protein family, implicated in this process by regulating and maintaining the structure of endothelial cell barrier function, and is vital in permeability changes [35]. VE-Cadherin mediates endothelial barrier dysfunction that contributes to the majority of CVD-related deaths associated with arteriosclerosis [36] by increasing endothelial permeability leading ultimately to endothelial failure. Reported studies indicated that TCDD, exogenous ligand of AHR, increased inflammatory responses through a nongenomic AHR function [37, 38] and led to functional activation of the tyrosine kinase Src by releasing from AHR complex. In search of potential AHR ligand, IS has been common characterstics with TCCD; however, response after IS activation of AHR on the physiological consequences and the underlying mechanisms are underexploited. Therefore, in this study, we provided evidence in IS activation of AhR and the effect on endothelial permeability using bovine aorta endothelial cells (BAECs). In our data, we found that IS increased AHR activation and endothelial cell permeability, which may contribute to developments of a cardiorenal syndrome and disease related to endothelial hyperpermeability pathologies under uremic condition. Taken together, the findings indicate that IS plays a pivotal role in the disruption of endothelial permeability via AHR/Src-dependent pathway and the pathologies mentioned above, and therefore, there is a pressing need for researchers to investigate compounds that can alleviate the deleterious effects of IS.

Resveratrol (trans-3,4,5-trihydroxystilbene, RES) is a natural polyphenolic phytoalexin compound found abundantly in grapes, berries, red wine, and various other dark skin fruits [39] and has potential uses in human medicine since it modulates numerous physiological functions and exerts multiple effects including cancer, neurological, hepatic, and cardiovascular disease management [40–42] and has pharmacological antagonism property of AHR [43]; it possesses antiatherosclerosis effect [44], ameliorates hyperpermeability induced by high glucose-mediated caveolae via VEGF/KDR [45], protects intestinal hyperpermeability, upregulates heme oxygenase-1 expression by lessening oxidative stress [46], and protects breakage of blood-brain barrier properties induced oxidized LDL [47]. Furthermore, RES has been documented as an inhibitor of Src tyrosine kinase activity and proved to decrease endothelial hyperpermeability [48]. Reported studies suggested that RES reduce aortic fatty streak accumulation, initial lesion for the progression of atherosclerosis [49]. RES may be effective as a protective agent against AHR ligands. Shreds of evidence from previous studies have shown that RES can compete with the ligand for AHR binding and efficiently block the induction of CYP1A1 by AHR ligand. Despite the fact that RES was shown to inhibit TCDD-activated AHR-induced CYP1A1 expression in HepG2 cells [50], its role in IS-activated AHR-induced endothelial permeability was rarely reported. As both IS and RES could interact with AHR, the quite different roles on health or diseases are of concern. Therefore, in this study, we explored IS effect on endothelial permeability and how RES can rescue IS-induced endothelial hyperpermeability using bovine aorta endothelial cells (BAECs) with and without RES. Our data showed that IS affect cell viability, disrupt TEER of the cell, and increased permeability by increasing the activity of AHR complex/Src signaling mediated VE-Cadherin. However, RES inhibit IS-induced activated Src signaling and VE-Cadherin and downregulated endothelial hyperpermeability via AhR/Src-dependent pathway. This study is of crucial importance in disease condition where endothelial permeability is compromised.

## 2. Materials and Methods

### 2.1. Reagent and Chemicals

We purchased bovine aortic endothelial cells (BAECs) from the Health Science Research Resources Bank (Osaka, Japan, no. C-003-5C). Dulbecco's modified Eagle's medium (DMEM) and Fetal Bovine Serum (FBS) were purchased from Gibco Inc. (Grand Island, NY, USA). FITC-dextran, PP2 (4 - Amino - 5 -( 4 - chlorophenyl )- 7 -( dimethylethyl ) pyrazolo [ 3 , 4 - d ]), (an inhibitor of Src), pSrc (phosphorylated Src) and Src, AHR inhibitor (CH223191), indoxyl sulfate (IS), and CCK-8 Kit were from Sigma-Aldrich China Inc. (Shanghai, China). AHR, ARNT, pVE-Cadherin (phosphorylated VE-Cadherin), and VE-Cadherin antibody were purchased from ABclonal (Wuhan, China); *β*-actin antibody was purchased from Sigma-Aldrich (St. Louis, MO, USA). Resveratrol (3,5,4′-trihydroxy-trans-stilbene) was purchased from Nanjing Zelang Biotechnology Inc., China.

### 2.2. Cell Culture

BAECs were maintained at 37°C in 5% CO_2_ in DMEM/low glucose containing 10% FBS, 100 IU/ml penicillin, and 100 *μ*g/ml streptomycin. Cells were used for experiments between passages 2 and 9. Confluent BAECs were treated with IS in the presence and absence of RES. Confluent cells without IS were used as a control group.

### 2.3. Cell Viability Assay

Cell viability was analyzed by the cell counting kit-8 (CCK-8) assay. First, the experiment was performed based on reported maximum serum IS concentration (about 200-250 *μ*g/ml) in uremic patients and exposed to the cells for different times to examine time-dependent effect of IS on the cells, and then, we make used of a wide range of concentration of IS for 24 h to determine the dose for further analysis. The cells were seeded in a 96-well plate at a density of 5 × 10^3^ cells per well.

After being treated by different concentrations, cells were washed with PBS three times and incubated with 90 *μ*l of fresh medium with 10 *μ*l CCK-8 solution at 37°C for 2 h. The optical density was measured by an enzyme-linked immunoassay analyzer at 450 nm.

### 2.4. Transepithelial Electrical Resistance (TEER) Measurement

Transepithelial/transendothelial electrical resistance (TEER) of the cell monolayer was determined by a Millicell ERS-2 system. TEER measurement is a widely recognized quantitative method to measure the resistance of cell monolayer and integrity of close-fitting junctional dynamics in cell culture models of endothelial and epithelial cells. BAECs were seeded in the upper chambers of polycarbonate Transwell inserts (0.4 *μ*M pore, 6.5 mm diameter, Millipore, USA) at 1.25 × 10^5^ cells/ml and cultured until confluent for 4 days. Groups of cells were treated with IS in the presence and absence of RES, AHR inhibitor (CH223191), and PP2 (Src inhibitor) for different times and compared with the control and treatment groups. The resistance of the monolayer was determined daily by measuring the TEER with a Millicell ERS-2 system. Cell resistance values of multiple Transwell inserts of the experimental group were measured sequentially, and the mean was expressed in *Ω* (Ohms) after subtraction of the value of a blank cell-free filter. The TEER of the monolayers was recorded when a stable resistance reading was achieved with triplicate measurements that were taken for each Transwell. The Millicell ERS-2 meter functionality was adjusted to ensure proper operation. The experiments were performed at 37°C with the passage cell numbers of 2 and 3. Resistance of the cell monolayer was calculated as per manufacturer instruction and considered as the area of the membrane used:

(1) $$\begin{array}{c} \text{Resistance Calculation}:R_{\text{cell monolayer}}=R_{\text{sample}}\text{R sample}-R_{\text{blank}}, \end{array}$$

where *R*_cell monolayer_ is resistance of the cell monolayer, *R*_sample_ is resistance reading across the cell (cell culture with cell), and *R*_blank_ is resistance reading across the blank cup (cell culture without cells).

Unit area resistance was also calculated as the value independent of the area of the membrane used; in this study, we used a 24-well plate: wall diameter 10 mm and membrane area 0.6 cm2:

(2) $$\begin{array}{c} \text{Unit Area Resistance}=\text{Resistance }\left(\Omega \right)\times \text{effective membrane area }\left({\text{cm}}^{2}\right)={\left(\Omega \text{cm}\right)}^{2}. \end{array}$$

### 2.5. Endothelial Permeability

Endothelial permeability was determined by the flux of FITC-labeled dextran tracers through the Transwell system. Briefly, BAECs were plated on the upper chambers of 0.4 *μ*m pore size Transwell at 1.25 × 10^5^ cells/ml and cultured until confluent. After treatment, the medium was replaced with fresh phenol red-free DMEM and FITC-dextran tracers (40 kDa, final concentration 1 mg/ml) were added into the upper chamber and incubated at 37°C for 2 h. After incubation, the amount of tracer in the lower chamber was determined by a microplate reader (Biotech, Highland Park, USA) at 494 nm excitation and 521 nm emission. All the data were recorded as the means of three experiments.

### 2.6. Western Blot Analysis

The expression level of proteins was analyzed using Western blot. Following IS treatment on BAECs in the presence and absence of RES and inhibitors, cells were washed with PBS and homogenized in 4°C radioimmunoprecipitation assay (RIPA) lysis buffer. Briefly, the cells were lysed in RIPA lysis buffer with phenylmethylsulfonyl fluoride (PMSF), placed on ice for 30 min, and centrifuged at 13500 rpm at 4°C for 15 minutes. The supernatant was collected, and protein concentration was quantified using a protein assay reagent (BCA Assay Kit). For the collection of cytosolic fractions of AHR, BAECs were suspended in ice-cold phosphate-buffered saline (PBS), homogenized for 10 strokes using a glass Dounce homogenizer, and centrifuged (12000 g, 30 s, 4°C). The pellet was collected and resuspended in 200 *μ*l of ice-cold buffer A (10 mM HEPES-KOH (pH 7.9), 1.5 mM MgCl_2_, 10 mM KCl, 0.5 mM dithiothreitol, 0.2 mM PMSF, 5 *μ*g/ml aprotinin, and 2 *μ*g/ml leupeptin). After incubation on ice for 10 min, 10 *μ*l of 10% IGEPAL was added, and the mixture was mixed thoroughly for 30 s and centrifuged (12000 g, 30 s, 4°C), and then, the supernatant was collected as the cytosolic fraction. Proteins were denatured and separated by sodium dodecyl sulfate polyacrylamide gel electrophoresis (SDS-PAGE) and transferred to poly(vinylidene fluoride) (PVDF) membranes. Then, membranes were blocked with 5% bovine serum albumin (BSA) for 1 h at room temperature. After blocking, membranes were washed with Tris-buffered saline with Tween 20 (TBST) three times for 10 minutes each time and incubated at 4°C overnight with specific primary antibodies such as VE-Cadherin (1 : 1000), pVE-Cadherin (1 : 1000), AHR (1 : 1000), Src (1 : 1000), pSrc (1 : 1000), *β*-actin (1 : 5000), and ARNT (1 : 1000). Then, membranes were washed with TBST for three times and incubated with secondary antibody for 1 h at room temperature. After washing in TBST, proteins were visualized by an enhanced chemiluminescence (ECL, Immobilon, USA) Western blotting system. The relative concentration of target protein expression was determined by computer analysis using ImageJ and normalized to an internal standard (*β*-actin).

### 2.7. Immunofluorescence Staining

BAECs were seeded and grown to confluence on fibronectin-coated glass chamber slides. After treatment, the medium was removed, and the monolayers were washed with PBS containing 100 ml-glycine, fixed with 4% paraformaldehyde, and washed three times with PBS for 10 min and blocked with 5% (*w*/*v*) BSA in PBS for 30 min. Immunofluorescence was performed by staining with primary antibody against AHR at a dilution of 1 : 400 overnight at 4°C. Specific binding was detected using conjugated goat anti-rabbit antibody followed by tyramine labeling with red fluorescent. The fluorescence images were picked up using a cell imaging system. The slides were photographed using an Olympus LCX200 Imaging System (Olympus Corporation, Tokyo, Japan).

### 2.8. Statistical Analysis

Data were presented as the *mean* ± *SEM* (*Standard* *Error* of the *Mean*) and analyzed using GraphPad Prism 7.0 for Windows-statistical software (GraphPad Prism software, La Jolla, California, USA, http://www.graphpad.com), and the statistical difference was determined by one-way ANOVA procedure followed by the Student-Newman-Keuls post hoc test with 95% confidence and unpaired *t*-test. The level of *P* < 0.05 was accepted as statistically significant.

## 3. Results

### 3.1. Effect of IS and RES on Cell Viability

**Figure 1:**
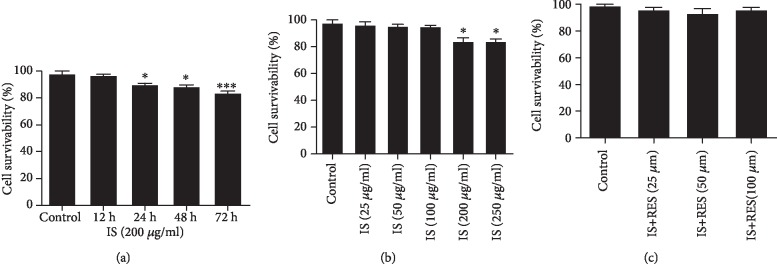
Effect of IS and RES on cell viability. (a) BAECs were incubated with IS (200 *μ*g/ml) for different times (12-72 h); cell viability was decreased in a time-dependent manner. (b) Cells were stimulated with various concentrations of IS (25-250 *μ*g/ml) for 24 h; the cell survivability also declined at high concentration. (c) RES treatment did not affect the viability of BAECs. Data are shown as the mean ± SEM. ^∗^*P* < 0.05 and ^∗∗^*P* < 0.01 vs. control.

The viability of BAECs was measured by using a CCK-8 uptake assay. First, BAECs were cultured and incubated with the maximum IS serum level under uremic condition (200 *μ*g/ml) for different times (12-72 h), and the result showed that the viability/survivability of BAECs was gradually declined in a time-dependent manner compared to the control (Figure 1(a)). Then, we have used a different concentration of IS (25-250 *μ*g/ml) and incubated for 24 h to determine the effect of IS, and cell survivability was declined significantly at high concentration of IS (200/250 *μ*g/ml) (Figure 1(b)); thus, we make used of 100 *μ*g/ml IS for subsequent experiments. Coincubation of different concentrations (25, 50, and 100 *μ*M) of RES did not affect the viability of BAECs (Figure 1(c)).

### 3.2. RES Supplementation Improved Impaired TEER Induced by IS

**Figure 2:**
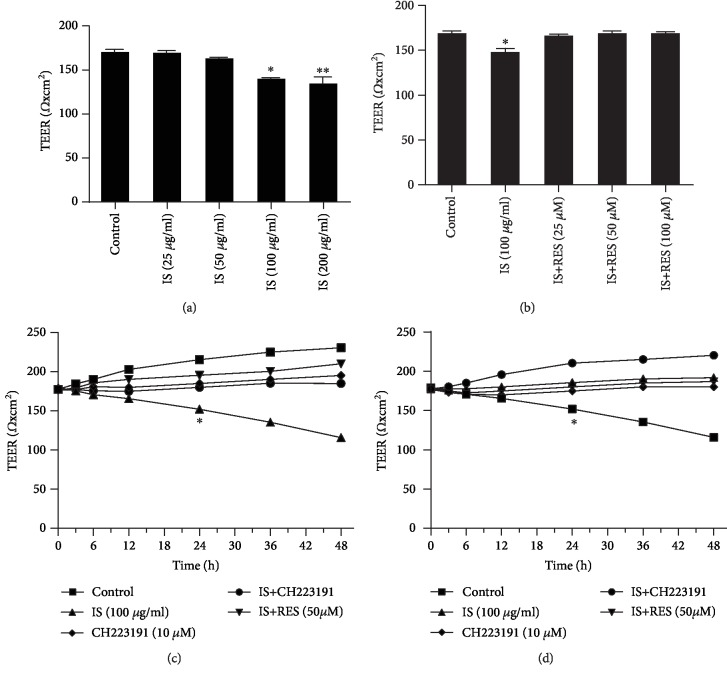
RES supplementation improved the impaired TEER induced by IS. (a) Millicell ERS-2 meter measurement result indicated that the increase in the concentration of IS treatment causes a gradual decrease in TEER value of cells compared to the control group. (b) RES pretreatment (25, 50, and 100 *μ*M) restored TEER of cells significantly. (c) TEER value of cells significantly decreased following IS treatment at 24 h, whereas there was no significant difference between the IS treatment group with CH223191 and RES compared with the control group. (d) Coincubation of IS with PP2 (10 *μ*M) also restored the decreased TEER of cells. Data are presented as the mean ± SEM. ^∗^*P* < 0.05 and ^∗∗^*P* < 0.01 vs. control; ^#^*P* < 0.05 vs. IS-treated group.

TEER of BAECs was measured by a Millicell ERS-2 system. TEER value is a strong indicator of cellular monolayer health and integrity in endothelial and epithelial monolayer cell culture models, which play a crucial role in regulating permeability. IS-induced disruption of endothelial cell monolayer integrity resulted in a sharp decrease in TEER of cells and increased permeability. Following incubation of cells with different concentrations of IS (25-200 *μ*g/ml) for 24 h, TEER value of cell monolayer was weakened and significantly reduced at 100 *μ*g/ml IS-incubated cells compared to the control (Figure 2(a)). RES supplementation at different concentrations (25, 50, and 100 *μ*M) significantly increased TEER of cells (*P* < 0.05) which was declined in response to IS (100 *μ*g/ml) exposure (Figure 2(b)). TEER value of cells also remained similar with the control following RES (50 *μ*M), CH223191 (10 *μ*M) pretreatment, and incubated cells with IS (100 *μ*g/ml) for different times, whereas a gradual decrease of TEER of cells was observed in IS (100 *μ*g/ml) treatment group (*P* < 0.05) (Figure 2(c)). Similar phenomena occurred in coincubation of PP2 (10 *μ*M) and IS (100 *μ*g/ml), and PP2 could restore the decreased TEER of cells (Figure 2(d)). Incubation of cells with CH223191 and PP2 did not affect the TEER of BAECs.

### 3.3. RES Attenuated IS-Increased Endothelial Permeability

**Figure 3:**
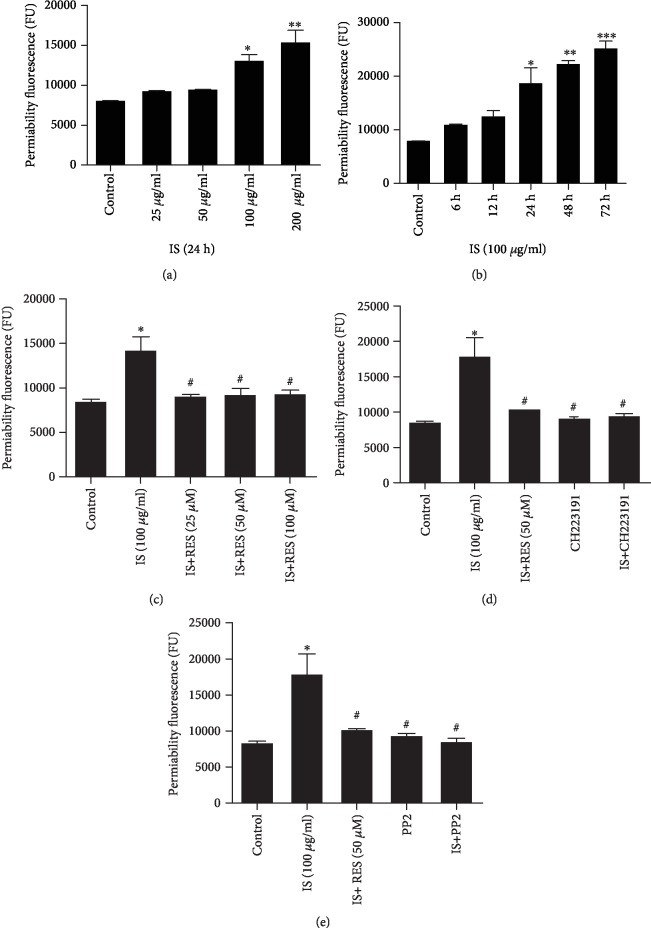
RES attenuated IS-increased endothelial cell permeability. (a) The permeability of endothelial cells gradually increased in response to different concentrations of IS for 24 h and reached significant at 100 *μ*g/ml of IS-treated cells. (b) Different incubation periods of IS (100 *μ*g/ml) increased cell permeability. (c) Cotreatment with RES (25, 50, and 100 *μ*M) decreased the hyperpermeability induced by IS. (d) Pretreatment of CH223191 and RES (50 *μ*M) markedly decreased permeability, respectively. (e) Coincubation of PP2 also decreased permeability. Data were expressed as the mean ± SEM. ^∗^*P* < 0.05, ^∗∗^*P* < 0.01, and ^∗∗∗^*P* < 0.001 vs. control; ^#^*P* < 0.05 vs. IS-treated group.

We then measured the fluorescence intensity flux of FITC-dextran, which passes through endothelial cells (Figure 3) to the lower chamber demonstrating the permeability. BAEC permeability was gradually increased with the increase in IS concentration (25-200 *μ*g/ml), and statistical significance reduction (*P* < 0.05) was observed in 100 *μ*g/ml IS treatment group (Figure 3(a)). Similarly, endothelial cell permeability was increased after incubation of cells (100 *μ*g/ml IS) for a different time in a time-dependent manner (Figure 3(b)). To determine the ameliorate property of RES on IS-induced permeability, BAECs were coincubated with different concentrations of RES (25, 50, and 100 *μ*M), and as expected, RES lead to a significant decrease in permeability (Figure 3(c)). Furthermore, pretreatment of cells with RES (50 *μ*M), CH223191 (10 *μ*M), and PP2 (10 *μ*M) decreased the hyperpermeability of cells induced by IS and brought close to the control level (Figures 3(d) and 3(e)), respectively. CH223191 and PP2 incubation on cells did not affect BAEC permeability.

### 3.4. IS Triggered AHR Activation and Increased Activation of Src and VE-Cadherin

**Figure 4:**
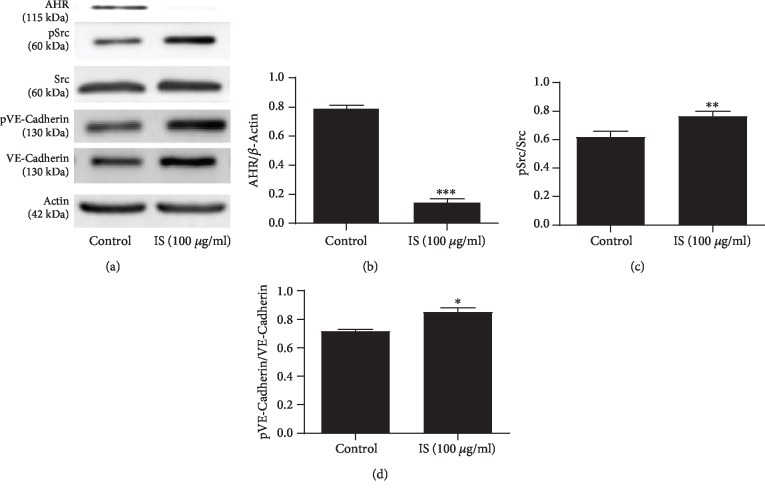
IS triggered AHR activation and increased activation of Src and VE-Cadherin. (a, b) Western blot analysis result showed that IS (100 *μ*g/ml) activated cytosolic AHR protein corresponding to the decreased cytosolic AHR (*P* < 0.001), (a, c) increased phosphorylation of Src, and (a, d) increased phosphorylation of VE-Cadherin protein, respectively (*P* < 0.01 and *P* < 0.05). Data are presented as the mean ± SEM. ^∗^*P* < 0.05, ^∗∗^*P* < 0.01, and ^∗∗∗^*P* < 0.001 vs. control.

Previous studies suggested that IS-induced activation of AhR leads to subsequent stimulation of downstream signal transduction to modulate protein and stimulates multiple pathways which lead to dysfunction of the endothelial cell [21, 51]. Since our previous data show that IS (100 *μ*g/ml) impaired TEER of cells and induced endothelial permeability, in this research, we investigated the underlying mechanism by making use of Western blot which revealed that incubation of cells with IS (100 *μ*g/ml) for 24 h triggered the activation of AHR and significantly decreased the cytosolic AHR protein expression level (Figures 4(a) and 4(b)) compared to the control which contributed to the increased activity of Src (phosphorylation of Src) (Figures 4(a) and 4(c)). Treatment of IS (100 *μ*g/ml) also significantly increased phosphorylation of Vascular Endothelial Cadherin protein (pVE-Cadherin) (Figures 4(a) and 4(d)), which may promote endothelial cell hyperpermeability.

**Figure 5:**
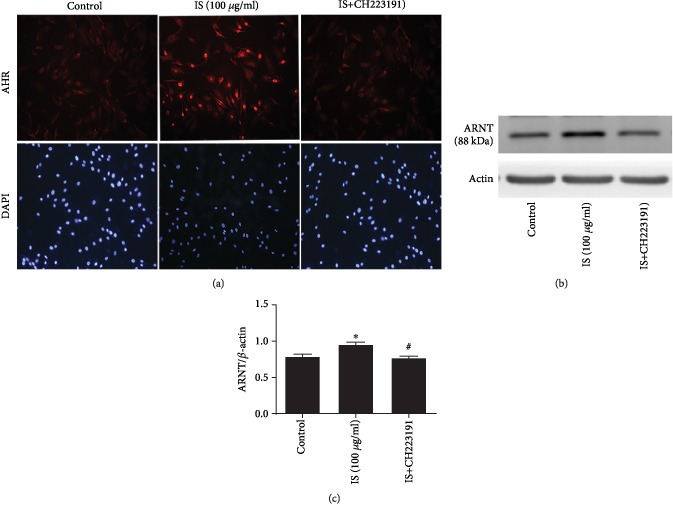
IS promotes AHR nuclear translocation and increased the level of ARNT expression level. (a) Immunofluorescence analysis using 200x objective results revealed the highly stained nuclear expression of AHR in the IS treatment group while treatment with CH223191 eliminated the effect of IS. (b, c) Western blot analysis also indicated that IS increased the expression of ARNT level while treatment with CH223191 decreased the expression of ARNT induced by IS. Data are expressed as the mean ± SEM. ^∗^*P* < 0.05 vs. control; ^#^*P* < 0.05 vs. IS-treated group.

Despite the fact that the AHR nongenomic pathway is ARNT-independent, in the presence of ligand, the AHR has been activated (translocated) and regulate gene expression through a nongenomic signaling pathway [26, 30] as AHR regulated the number of genes without direct binding of the AHR to DRE consensus sequence [31]. Thus, in order to confirm the potential of IS on AHR activation, we determined the expression level of ARNT using Western blot; at the same time, we make used of immunofluorescence to observe the nuclear expression of AHR where BAECs were stained with anti-AHR antibody (red) and DAPI (blue) and visualized by fluorescence microscopy revealing a highly stained nuclear expression of AHR in IS treatment compared with the control while treatment with CH22319, an inhibitor of AHR, prevented the activation of AHR (Figure 5(a)). Simultaneously, we carried out Western blot analysis and observed a significantly increased (*P* < 0.05) ARNT (aryl hydrocarbon receptor nuclear translocator) protein expression level in response to IS treatment compared with the control (Figures 5(b) and 5(c)) which confirmed the direct consequence of IS on cytosol AhR activation.

### 3.5. Involvement of Src Kinase in AhR-Mediated Modulation of Permeability in response to IS Exposure

**Figure 6:**
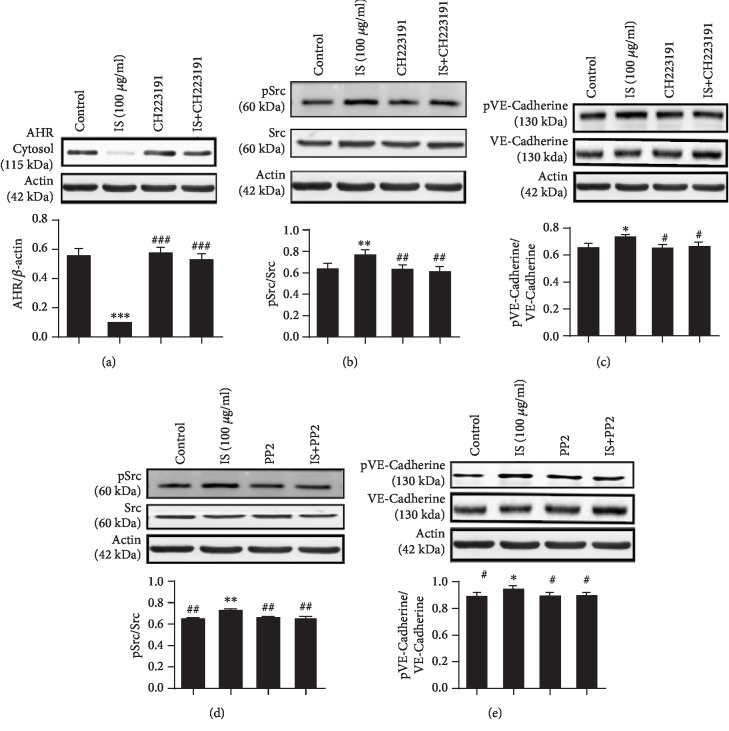
Src kinase involved in AhR-mediated modulation of permeability in response to IS exposure. (a) Cytosolic AHR expression level significantly decreased in response to IS exposure; cotreatment with CH223191 increased cytosolic AHR expression level. (b) CH223191 decreased Src phosphorylation significantly induced by IS. (c) CH223191 also reduced the increased activity of VE-Cadherin induced by IS. (d) PP2 abolished Src phosphorylation induced by IS, and (e) PP2 significantly decreased VE-Cadherin phosphorylation induced by IS. Data are presented as the mean ± SEM. ^∗^*P* < 0.05, ^∗∗^*P* < 0.01, and ^∗∗∗^*P* < 0.001 vs. control; ^#^*P* < 0.05, ^##^*P* < 0.05, and ^###^*P* < 0.01 vs. IS-treated group.

Several studies that reported using prototypic AhR agonists such as TCDD have led to the introduction of AHR-dependent Src kinase activation pathways, and recently, some of the toxic effects of these environmental pollutants are known [22]. Evidence indicated that Src protein kinase is located in the cytosol of cells complexed with AhR, and upon ligand binding with the AHR subunit, the Src kinase is released from the AHR complex and translocated to the cell membrane and regulates permeability. Our present data also revealed that IS activated AHR (decreased cytosolic AHR expression level) (*P* < 0.001); however, coincubation with AHR blocker, CH223191 (10 *μ*M), inhibits the activation of cytosolic AHR (Figure 6(a)). IS causes a considerable rise in phosphorylation of Src (*P* < 0.01), and by making use of AHR blocker, CH223191 (10 *μ*M), we also provided evidences that the activation and involvement of Src in permeability change in response to IS exposure are directly correlated with activated AHR by IS because CH223191 significantly eliminated the increased activity (phosphorylation of Src) (Figure 6(b)) (*P* < 0.05) and VE-Cadherin; the phosphorylation also decreased after incubation of the cell with CH223191 (*P* < 0.05) (Figure 6(c)) which may contribute to decreasing endothelial permeability induced by IS. Src inhibitor, PP2 (10 *μ*M), also markedly abolished phosphorylation of Src (Figure 6(d)), and decreased VE-Cadherin phosphorylation induced by IS-activated AHR (Figure 6(e)) may lead to a decrease in endothelial permeability.

### 3.6. RES Inhibits Src Kinase Activity Mediating VE-Cadherin Induced by IS-Activated AHR and Modulates Permeability via AHR

**Figure 7:**
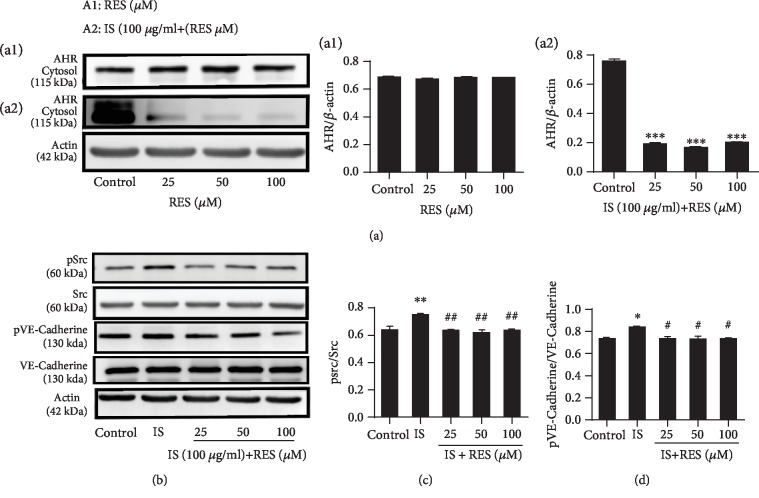
RES inhibits Src kinase activity mediating VE-Cadherin activity induced by IS-activated AHR and modulates permeability via AHR: (a1) RES (25, 50, and100 *μ*M) did not affect cytosolic AHR protein expression level; (a2) cytosolic AHR protein decreased significantly following coincubation of IS with RES (b, c). RES could significantly decrease the Src phosphorylation (b, d) RES also decreased the activity of VE-Cadherin induced by IS (100 *μ*g/ml). Data were expressed as the mean ± SEM. ^∗^*P* < 0.05, ^∗∗^*P* < 0.01, and ^∗∗∗^*P* < 0.001 vs. control; ^#^*P* < 0.05, ^##^*P* < 0.01, and ^###^*P* < 0.001 vs. IS-treated group.

In our study, first, we examined the role of RES on cytosolic AHR with or without the administration of IS. The Western blot analysis result demonstrated that the cytosolic AHR protein expression level was not affected by RES treatment at different concentrations (25, 50, and 100 *μ*M) (Figure 7(a), a1). However, the loss of the cytosol AHR protein expression level was observed in the coincubation of RES with IS (100 *μ*g/ml) (Figure 7(a), a2). Treatment of cells with different concentrations of RES (25, 50, and 100 *μ*M) significantly reduced the Src phosphorylation induced by IS (*P* < 0.05) (Figures 7(b) and 7(c)). RES also significantly reduced VE-Cadherin phosphorylation with a statistical significance (*P* < 0.05) increased by IS and modulate endothelial permeability (Figures 7(b) and 7(d)).

## 4. Discussion

Our findings illustrated that the upregulation of IS activated the AHR/Src/VE-Cadherin pathway in hyperpermeability of bovine aorta endothelial cells ameliorated by RES and this is of relevance in CDK condition where upregulation of AHR/Src/VE-Cadherin activity in the pathogenesis of endothelial hyper-permeability-like pathology under uremic condition. Moreover, due to multiple biological function of RES and good permeation characteristics in endothelial cell with low toxicity even in high dose showed promising virtue. Previous studies demonstrated that due to deterioration of renal filtration in CKD patients, IS level in serum is elevated and develops a major risk factor for atherosclerosis [52, 53]. Since endothelial hyperpermeability is the result of endothelial dysfunction which is a marker of the early stage of atherosclerosis, inhibition of IS-induced endothelial hyperpermeability will play beneficial roles in maintaining endothelial cell function and prevent the development of atherosclerosis under uremic condition. Research studies reported that RES improves endothelial function and has beneficial effects on vascular tone and vessels in human and animal models [54]. Therefore, we investigated the effect of IS on BAECs and the role of RES against IS-induced endothelial hyperpermeability in this study.

When the cytotoxicity test for BAECs was investigated, pernicious effects of IS on cell barrier dysfunction were observed in cultured human umbilical vascular endothelial cells (HUVECs) [52]; in humans, they have also been associated with various adverse outcomes such as renal disease and vascular and bone diseases [55]. Even under the pathological condition, the increased plasma level of IS induced oxidative stress, and this event leads to cellular toxicity by [48]. In this perspective, our present study highlights cytotoxicity of IS, which indicates a time- and dose-dependent pattern suggesting its toxicity (Figure 1). Since the cell viability demonstrated a gradual decrease, IS inhibit the viability of cells significantly at high concentration (200/250 *μ*g/ml)-incubated cells for 24 h (Figure 1(a)). Our data are comparable with multiple studies about IS renal and nonrenal effects [9, 55]. Thus, we used 100 *μ*g/ml of IS for the remaining experiments which is compatible with the serum concentration level in uremic condition. Treatment of different concentrations of RES (25, 50, and 100 *μ*M) did not affect the viability of BAECs (Figure 1(c)).

Subsequently, the Millicell ERS-2 meter and flux of FITC-labeled dextran tracer assay were used to examine IS effect on TEER and permeability of BAECs, respectively, and evaluated the effect role of RES supplementation. The greater TEER value of cells is an indicator of cell monolayer health, confluence, structural barrier integrity, and the lesser endothelial cell hyperpermeability. In line with previous studies where IS mediated increase in endothelial permeability by inducing endothelial barrier dysfunction, disrupting intercellular junctions, and inducing intercellular gap formation [56], similar phenomena were observed in the present study (Figure 2) where IS decreased TEER value of BAECs (Figure 2) suggesting the increase in concentration of IS incubation that resulted in weakened cell monolayer which leads to an increased endothelial permeability. Treatment with RES (25, 50, and 100 *μ*M) rescued TEER of cell impairment and restored significantly suggesting its potential of repairing injured cell monolayer and maintaining endothelial cell membrane disruption induced by IS and decreasing endothelial hyperpermeability. The longer incubation period of cell with 100 *μ*g/ml of IS also decreased TEER value of cells and contributed for increasing endothelial permeability while in the opposite, TEER value of cells coincubated with RES (50 *μ*M), CH223191 (10 *μ*M), and PP2 (10 *μ*M), respectively, had been comparable with the control group suggesting their ameliorative property. The findings are in agreement with the previous study showing its potential. It has been reported that RES has the potential to improve the release of nitric oxide (NO) and prostacyclin (PGI), thus playing a vital role in the maintenance of endothelial function [54]. RES also, as documented by some studies, acts as an inhibitor of Src tyrosine kinase activity and has been proved to decrease endothelial hyperpermeability [48]. The potential of PP2 to inhibit the increase in pulmonary microvascular leakage was also reported implying its action as a potent downregulator of endothelial hyperpermeability [57]. Furthermore, studies revealed that CH223191 also could inhibit migration of cells in response to TCDD-activated AhR in [58]. To further support our findings, we carried out flux FITC-labeled dextran tracer assay and measured the endothelial permeability, which revealed similar phenomena with the result found in TEER (Figure 3). CH223191 and PP2 treatment did not affect TEER of BAEC monolayer and permeability. Given the effect mentioned above of IS on endothelial permeability and ameliorative property of RES, AHR, and Src blockers in alleviating endothelial hyperpermeability, we make use of Western blot to assess the potential mechanisms of AHR/Src pathways being involved in endothelial permeability.

In nongenomic AHR pathway, ligand binding to AHR promotes Src kinase dissociation from AHR complex in the cytoplasm and leads to cell membrane translocation; thus, Src activity is increased and promoting disruption of cell-cell contact. Several studies have reported that the activity of Src can be upregulated by phosphorylation at Tyr 416, situated present in the catalytic domain in response to stimulation of a variety of cellular receptors, and regulate different signaling pathways and control different receptor-induced biological activities [59]. In the present study, our Western blot analysis result demonstrated that loss of cytosolic AHR protein expression was observed following IS treatment suggesting that IS could trigger the activation of AHR and nuclear translocation in the meantime and promote the release of Src from its receptor complex in the cytoplasm and increased Src activity; in line with this, IS causes a significant increase of Src phosphorylation which mediates the most significant permeability-associated protein (VE-Cadherin) activity and may lead to the increased endothelial hyperpermeability (Figure 4). VE-Cadherin is known to mediate endothelial permeability modulation [57], and numerous stimuli may cause VE-Cadherin phosphorylation, in which Src kinase involves and acts as a key pathway mediator. Here, in this setting, we noticed increased phosphorylation of VE-Cadherin following IS treatment suggesting an increase in endothelial permeability.

As shown in Figure 5(a), the immunofluorescence analysis result further supported the activation and AHR nuclear translocation in response to IS exposure of cells stained with anti-AHR antibody (red) and DAPI (blue) and visualized by fluorescent microscopy which demonstrated highly stained nuclear expression of AHR and an increase in fluorescence intensity in the IS treatment group compared with the control whereas pretreatment of cells with AHR inhibitor (CH223191) blocked the activation and AHR nuclear translocation induced by IS (Figure 5(a)). Western blot analysis also indicated that an increased expression of ARNT is associated with activation and AHR nuclear translocation while treatment with AHR inhibitor (CH223191) decreased the expression level of ARNT induced by IS (Figures 5(b) and 5(c)), and this further confirmed the direct consequence of IS on AHR activation and promoting AHR nuclear translocation from the cytosol compartment to the nucleus and may lead to forming heterodimeric with ARNT and contribute to the increased expression level of ARNT.

Studies indicated that the increased activation of Src kinase following ligand-activated AhR has been noted in several different cell lines and hence may be essential for AhR-mediated regulation of AR activity [60]. Evidence suggesting that activation of AHR may result in deregulation of cell-cell contact has been put forth, hence evoking unbalanced proliferation and dedifferentiation, as well as enhanced motility [61]. Src kinase which has been associated with AHR complex is important in the disruption of cadherin-dependent cell-cell contact. Here, to confirm the involvement of Src kinase on AHR mediating endothelial permeability in response to IS exposure, we incubated cells with AHR blocker (CH223191) with IS (100 *μ*g/ml) and employed Western blot. The result demonstrated that IS increased AHR activation and CH223191 abolished AHR activation (Figure 6(a)). IS also triggered Src activation; however, CH223191 inhibit its activation (Figure 6(b)) and reduced phosphorylation of VE-Cadherin induced by IS (Figure 6(c)). From this event, we suggested that the inhibitions of Src phosphorylation are AHR-dependent because CH223191 significantly eliminated phosphorylation of Src and may contribute to the reduction of VE-Cadherin activity and suggested that Src kinase activity is essential for regulating endothelial permeability via AHR. The result is comparable with studies demonstrated where Src activation could also contribute to VE-Cadherin phosphorylation by stimulation of H_2_O_2_ [2]. From previous studies, it was reported that Src mediates vascular endothelial permeability due to TNF, reactive oxygen species (ROS), angiogenesis, and vascular leakage [59]; therefore, in this manner, the IS acts AHR activation and lead to upregulate the activity of Src which mediate the activity of VE-Cadherin, therefore increasing endothelial hyperpermeability. Similar phenomena were observed where treatment of cells with Src blockers abolished Src phosphorylation (Figure 6(d)) and VE-Cadherin phosphorylation (Figure 6(e)) induced by IS implying that PP2 has a potential to inhibit the increased activity of Src mediating VE-Cadherin and reduce hyperpermeability induced by IS. These results are also in agreement with studies which reported that phosphorylation of Src gives rise to endothelial hyperpermeability due to stimulation by Advanced Glycation End-products (AGEs) and PP2 downregulated Src expression illustrating that inactivation of Src decreased AGE-induced HUVEC proliferation, migration, and tube formation [62]. Next, we observed the effect of RES on cytosolic AHR and then evaluated coincubation with IS (Figure 7). The data demonstrated that treatment of cells with RES did not affect the cytosolic AHR protein level. However, loss of cytosolic AHR was observed following coincubation of IS with RES implying that IS aggravated the activation of AHR and promote nuclear translocation from the cytosolic compartment. In this setting, RES did not inhibit or interfere IS binding/activating AHR in the cytoplasm. In the present study, the role of RES illustrating inhibition of Src activation induced IS-activated AHR and modulated VE-Cadherin and permeability. Controversial study results have been reported with regard to RES inhibitory property of ligands activating AHR. Few reports revealed that RES prevent ligand AHR activation by interfering ligand binding with AHR in the cytoplasm whereas the activity of RES may occur in the nucleus after nuclear translocation AHR induced by ligands. In the present study, our finding is correlated with the previous study, where RES interferes with Src tyrosine kinase activity [47]. Our data is comparable with studies reported on the inhibitory property of resveratrol on TCDD-induced CYP1A1 expression in HepG2 cells and the inability of RES to bind AHR and interfere the binding of AHR with TCCD [50]. Here, our data (Figure 7) also revealed that RES markedly decreased phosphorylation of Src induced by IS-activated AHR and downregulate the activity of VE-Cadherin and decreased hyperpermeability significantly thus implying that AHR/Src kinase is necessary for IS-mediated stimulation of permeability-associated protein, VE-Cadherin activity. These observations strongly indicate a possible connection between ligand-activated AHR, Src kinase, and VE-Cadherin. Indeed, our current data show that blockage of Src activity by resveratrol would IS-induced tyrosine phosphorylation of VE-Cadherin and the subsequent decreased endothelial cell permeability. The inhibition of RES on ligand-AHR activation and its biological effect in the nucleus and cytoplasm (genomic and nongenomic AHR pathway) require further investigation.

## 5. Conclusions

All our data demonstrated that AHR/Src-dependent-driven pathways are involved in IS-induced hyperpermeability of BAECs. TEER of BAECs were weakened in response to IS stimulation which leads to increased permeability, and the possible underlying mechanism could be that IS activated AHR/Src signaling which mediates endothelial permeability via the most important permeability-associated protein, VE-Cadherin. RES improved TEER of cells, suppressed IS-stimulated endothelial hyperpermeability, and downregulated the increased activity of VE-Cadherin, possibly by inhibition of the activation Src induced by IS-activated AHR. Altogether, our findings show in part evidence of the protective property of RES via blockade of IS-AHR mediating Src activation and downregulating the activity of VE-Cadherin and hyperpermeability.

## Data Availability

The data used to support the finding of this study are included in the article and can be acquired from the corresponding author.

## Abbreviations

AHR: Aryl hydrocarbon receptor
ARNT: Aryl hydrocarbon nuclear translocator
BAECs: Bovine aorta endothelial cells
BSA: Bovine serum albumin
CCK-8: Cell counting kit-8
CKD: Chronic kidney disease
CVD: Cardiovascular disease
DMEM: Dulbecco's modified Eagle's medium
ECL: Enhanced chemiluminescence
FBS: Fetal bovine serum
IS: Indoxyl sulfate
OD: Optical density
PBS: Phosphate-buffered saline
PMSF: Phenyl-methane-sulfonyl fluoride
PP2: 4-Amino-5-(4-chlorophenyl)-7-(dimethylethyl)pyrazolo[3,4-d]pyrimidine
PVDF: Polyvinylidene difluoride
RES: Resveratrol
RIPA: Radioimmunoprecipitation assay SDS-PAGE
Electrophoresis: Sodium dodecyl sulfate polyacrylamide gel
SEM: Standard error of the mean
pSrc: Phosphorylated Src
TBST: Tris-buffered saline with Tween
TCDD: Tetrachlorodibenzo-p-dioxin
TEER: Transendothelial electrical resistance
VE-Cadherin: Vascular endothelial cadherin
pVE-Cadherin: Phosphorylated vascular endothelial cadherin.
